# Persistent viral infections and their role in heart disease

**DOI:** 10.3389/fmicb.2022.1030440

**Published:** 2022-11-24

**Authors:** Ashwin Badrinath, Sagar Bhatta, Anna Kloc

**Affiliations:** Department of Biology and Environmental Science, University of New Haven, West Haven, CT, United States

**Keywords:** myocarditis, dilated cardiomyopathy, SARS-CoV-2, herpesvirus, persistent viral infection, adenovirus, coxsackievirus, parvovirus B19

## Abstract

Viral infections are the culprit of many diseases, including inflammation of the heart muscle, known as myocarditis. Acute myocarditis cases have been described in scientific literature, and viruses, such as parvovirus B19, coxsackievirus B3, or more recently, SARS-CoV-2, were the direct cause of cardiac inflammation. If not treated, myocarditis could progress to dilated cardiomyopathy, which permanently impairs the heart and limits a person’s lifespan. Accumulated evidence suggests that certain viruses may persist in cardiac tissue after the initial infection, which could open up the door to reactivation under favorable conditions. Whether this chronic infection contributes to, or initiates, cardiac damage over time, remains a pressing issue in the field of virus-induced heart pathology, and it is directly tied to patients’ treatment. Previously, large case studies found that a few viruses: parvovirus B19, coxsackievirus, adenovirus, human herpesvirus 6, cytomegalovirus and Epstein–Barr virus, are most commonly found in human endomyocardial biopsy samples derived from patients experiencing cardiac inflammation, or dilated cardiomyopathy. SARS-CoV-2 infection has also been shown to have cardiovascular consequences. This review examines the role of viral persistence in cardiac inflammation and heart disease, and discusses its implications for patients’ outcomes.

## Introduction

**Figure 1:**
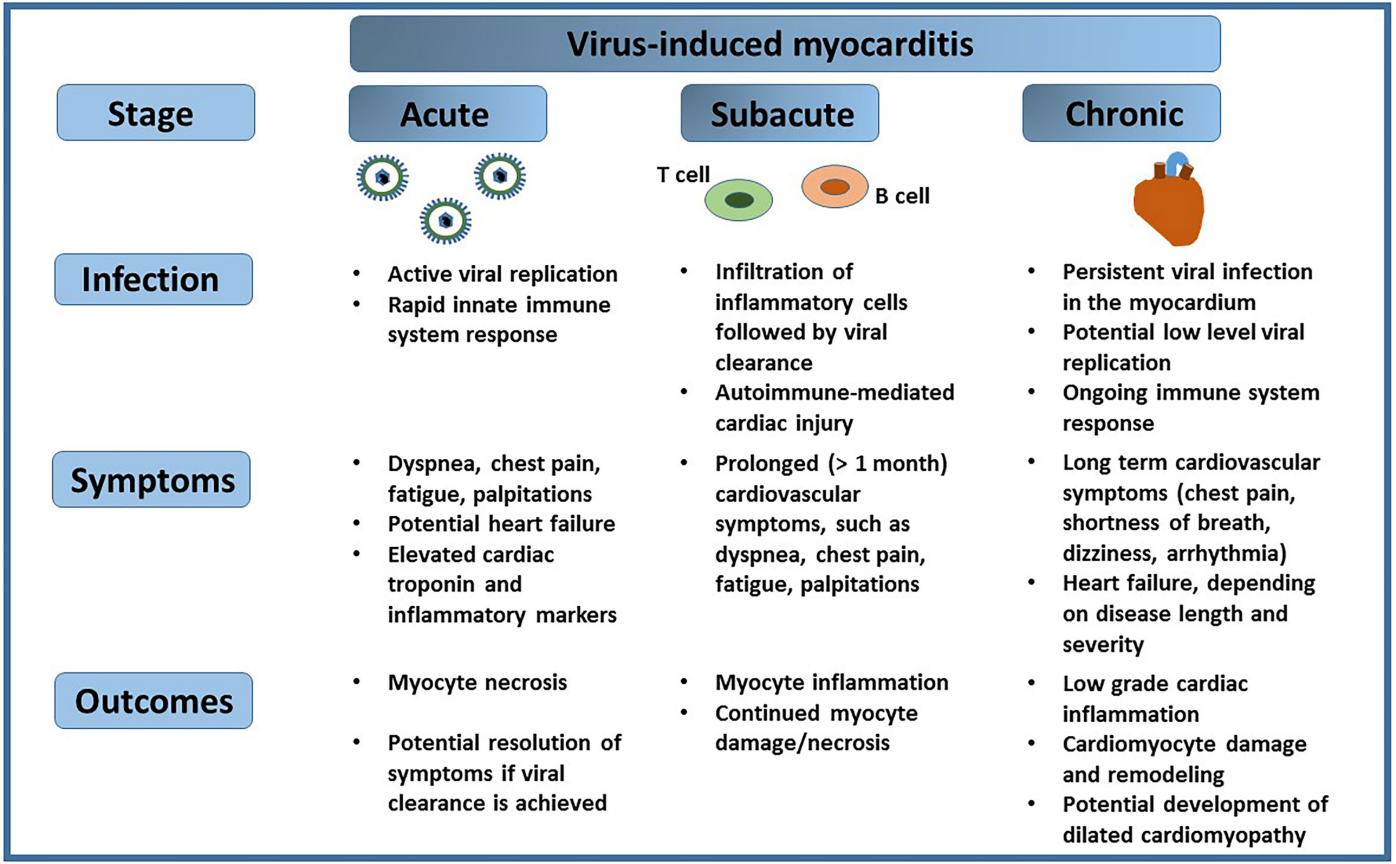
Virus-induced myocarditis. A viral infection of the heart can progress from the acute stage, manifested by rapid viral replication, to a chronic stage, which may involve periods of viral reactivation followed by silent infection. Each of the stages of viral infection of the cardiac muscle may present specific symptoms, and result in outcomes that impact cardiovascular health.

The cardiac muscle, or myocardium, is responsible for pumping blood throughout the cardiovascular system. Disease of the cardiac muscle, broadly defined as cardiomyopathy, can induce pathological changes to the muscle itself and negatively affect its function. The most common types of cardiomyopathy include dilated cardiomyopathy (DCM), hypertrophic cardiomyopathy, restrictive cardiomyopathy, arrhythmogenic cardiomyopathy or Takotsubo cardiomyopathy [reviewed in Brieler et al. (2017); Ciarambino et al. (2021)]. Inflammatory cardiomyopathy of the cardiac muscle, defined as myocarditis, is primarily triggered by viruses, although it can also be initiated by bacteria, fungi or parasites, or it may be a result of an autoimmune response. Occasionally, myocarditis can be caused by toxins or drugs, or it can be idiopathic in nature [reviewed in Tschöpe et al. (2021)]. The American Heart Association (AHA) defines myocarditis as inflammation of the heart muscle, and it estimates that the incidence of myocarditis in a general population is 22 in 100,000, or 0.022%. The disease is characterized by different stages, each with variable degree of severity, whereas a clinical diagnosis of myocarditis distinguishes an acute and a chronic (persistent) type. Acute myocarditis is often a consequence of a recent viral infection (Cooper, 2009), and symptoms associated with the disease, such as dyspnea, chest pain, fatigue, or palpitations, last from a few weeks to a few months (Dennert et al., 2008; Cooper, 2009). On the other hand, fulminant myocarditis, a subtype of acute myocarditis, is characterized by its sudden onset and rapid progression, along with extensive cardiac inflammation (Veronese, 2018). Activation of the adaptive immune system response in the subacute stage of myocarditis initiates viral clearance. However, after the initial infection subsides, the virus may persist in the heart tissue for an extended period of time and potentially cause direct injury to cardiomyocytes, or indirect damage to the heart tissue through chronic inflammation. Persistent viral infection of the cardiac muscle can involve intermittent stages of viral reactivation, followed by silent infection when no viral activity may be detected, or continuous replication of viral genome (Pollack et al., 2015; Tschöpe et al., 2021). Both processes could have a long lasting impact on cardiovascular health (Figure 1).

Acute and persistent myocarditis are classified based on the Dallas criteria (Thomas, 1987). The heart pathology associated with acute viral infection can be confirmed microscopically and it requires an endomyocardial biopsy (EMB). In such case, the histopathological staining reveals lymphocytic infiltrates and myocyte necrosis, consistent with injury of cardiac cells (Thomas, 1987). Direct injury of cardiomyocytes occurs during the course of viral infection; however, the host immune system response to the infection can further damage the cardiac cells. For example, in a mouse model of coxsackievirus B3 (CVB3) myocarditis, CD8^+^ cytotoxic T lymphocytes, which belong to the adaptive immune system, lyse the cardiomyocytes infected by the virus (Henke et al., 1995; Yajima, 2011). Although this response is critical for viral clearance, an enhanced immune system reaction to the infection may further exacerbate cardiac damage. If acute myocarditis is not fully resolved, persistent viral infection induces an inflammatory process that causes injury to cardiomyocytes, and ultimately leads to permanent cardiac muscle remodeling (Yajima, 2011).

DCM can be a potential outcome of unresolved myocarditis, and it is estimated that 20% of clinically proven myocarditis cases progress to DCM (Tschöpe et al., 2019). In humans, the characteristic features of DCM include a decrease in systolic ventricular function combined with ventricular chamber size enlargement. Patients suffering from DCM often develop chronic heart failure, which significantly limits their lifestyle. In the absence of a heart transplant option, more than 50% of individuals die within 5 years after the initial disease diagnosis (Bytyçi and Bajraktari, 2015).

## Myocarditis diagnosis

Clinical evaluation of patients’ symptoms is critical for disease diagnosis. Individuals suffering from heart inflammation often experience arrhythmias, chest pain or dyspnea, and they usually present with abnormal ECG (Pollack et al., 2015). Blood tests from patients suspected of acute myocarditis often reveal upregulated cardiac enzymes, such as troponin and N-terminal pro-B-type natriuretic peptide (Siripanthong et al., 2020). Neutralizing antibody titers were also previously measured in patients suffering from myocarditis and pericarditis (Fairley et al., 1996). However, to date, EMB with immunohistochemistry remains the golden clinical tool that allows to definitively confirm the presence of viral genome(s) in the heart samples, and to potentially link it to cardiac inflammation (Hazebroek et al., 2014). The procedure carries a low risk of complication, such as cardiac perforation, and it is not recommended for routine examination of heart related issues, or in a case of new onset of heart disease, which could potentially lower the number of known viral myocarditis cases.

Numerous viruses have been associated with cardiac inflammation (Table 1; Galpine and Wilson, 1959; Ainger et al., 1966; Maller et al., 1967; Mahapatra and Ellis, 1985; Özkutlu et al., 1989; Thanopoulos et al., 1989; Tsintsof et al., 1993; Martin et al., 1994; Schowengerdt et al., 1997; Fukae et al., 2000; Ishikawa et al., 2005; Matsumori and Hepatitis, 2005; Saggioro et al., 2007; Chapman et al., 2008; Kuchynka et al., 2010; Treacy et al., 2010; Leveque et al., 2011; Mutlu et al., 2011; Ukimura et al., 2012; Vanstechelman and Vandekerckhove, 2012; Donoiu and Istrătoaie, 2013; Jain et al., 2013; Kalimuddin et al., 2013; Wu et al., 2013; Tsuzuki et al., 2014; Gülhan et al., 2015; Premkumar et al., 2015; Aletti et al., 2016; Li et al., 2016; Ntusi et al., 2016; Chertow et al., 2017; Milas et al., 2017; Ntusi, 2017; Allen et al., 2018; Roto et al., 2018; Saraca et al., 2018; Wiyatno et al., 2018; Yamamoto et al., 2018; Farias et al., 2019; Paixão et al., 2019; Ackermann et al., 2020; Fox et al., 2020; Diarra et al., 2021; Formisano et al., 2021; Halushka and Vander Heide, 2021; Obrien et al., 2021; Cebeci et al., 2022; Colombo et al., 2022; Jumah et al., 2022). Some of them, such as parvovirus B19 or CVB3, are common among individuals in a general population and usually cause a mild illness, yet occasionally, they trigger cardiac inflammation (Jain et al., 2013; Wu et al., 2013). Rare, but severe diseases, such as Ebola or Hantavirus Pulmonary Syndrome (HPS), have also been reported to cause cardiac inflammation, albeit in sporadic cases (Saggioro et al., 2007; Chertow et al., 2017). More recently, SARS-CoV-2, a virus that emerged in late 2019, has been associated with myocarditis and cardiovascular outcomes (Fox et al., 2020; Halushka and Vander Heide, 2021; Jumah et al., 2022). Cardiomyopathy associated with viral reactivation may also occur in response to immune suppression after chemotherapy treatment (Varkoly et al., 2022).

**Table 1:** Viruses previously reported to cause myocarditis.

Viral genome type	Virus	References
ssDNA	Parvovirus B19 (B19V)	Schowengerdt et al. (1997); Jain et al. (2013); Ackermann et al. (2020)
dsDNA	Adenovirus	Martin et al. (1994); Treacy et al. (2010)
	Cytomegalovirus (HHV-5, CMV)	Vanstechelman and Vandekerckhove (2012); Saraca et al. (2018)
	Epstein-Barr virus (HHV-4)	Ishikawa et al. (2005); Mutlu et al. (2011)
	Herpesvirus 1 & 2	Kuchynka et al. (2010); Yamamoto et al. (2018); Colombo et al. (2022)
	Hepatitis B	Mahapatra and Ellis (1985)
	Human herpesvirus 6 (HHV-6)	Fukae et al. (2000); Leveque et al. (2011)
	Trichodysplasia spinulosa-associated polyomavirus (TSV)	Tsuzuki et al. (2014)
	Varicella-Zosten virus	Tsintsof et al. (1993); Donoiu and Istrătoaie (2013)
(+) ssRNA	Coxsackievirus	Chapman et al. (2008); Wu et al. (2013); Diarra et al. (2021)
	Chikungunya virus	Alvarez et al., 2017; Farias et al. (2019)
	Dengue virus	Li et al. (2016); Farias et al. (2019)
	Echovirus	Maller et al. (1967)
	Hepatitis A	Allen et al. (2018)
	Hepatitic C	Matsumori and Hepatitis (2005)
	Hepatitis E	Premkumar et al. (2015)
	Human immunodeficiency virus 1&2 (HIV)	Ntusi et al. (2016); Ntusi, 2017
	Human rhinovirus	Wiyatno et al. (2018); Cebeci et al. (2022)
	Poliovirus	Galpine and Wilson (1959)
	Rubella virus	Ainger et al. (1966); Thanopoulos et al. (1989)
	SARS-CoV-2	Fox et al. (2020); Halushka and Vander Heide (2021); Jumah et al. (2022)
	Yellow fever virus	Paixão et al. (2019)
	Zika virus	Aletti et al. (2016)
(–) ssRNA	Crimean-Congo hemorrphagic fever virus	Gülhan et al. (2015)
	Ebola virus	Chertow et al. (2017)
	Hantavirus	Saggioro et al. (2007)
	Human parainfluenza virus (HPIV)	Kalimuddin et al. (2013); Formisano et al. (2021)
	Influenza A & B	Ukimura et al. (2012); Roto et al. (2018)
	Mumps virus	Özkutlu et al. (1989)
	Respiratory syncytial virus (RSV)	Milas et al. (2017); Obrien et al. (2021)

## Viral infection of the heart – A question of persistence

**Figure 2:**
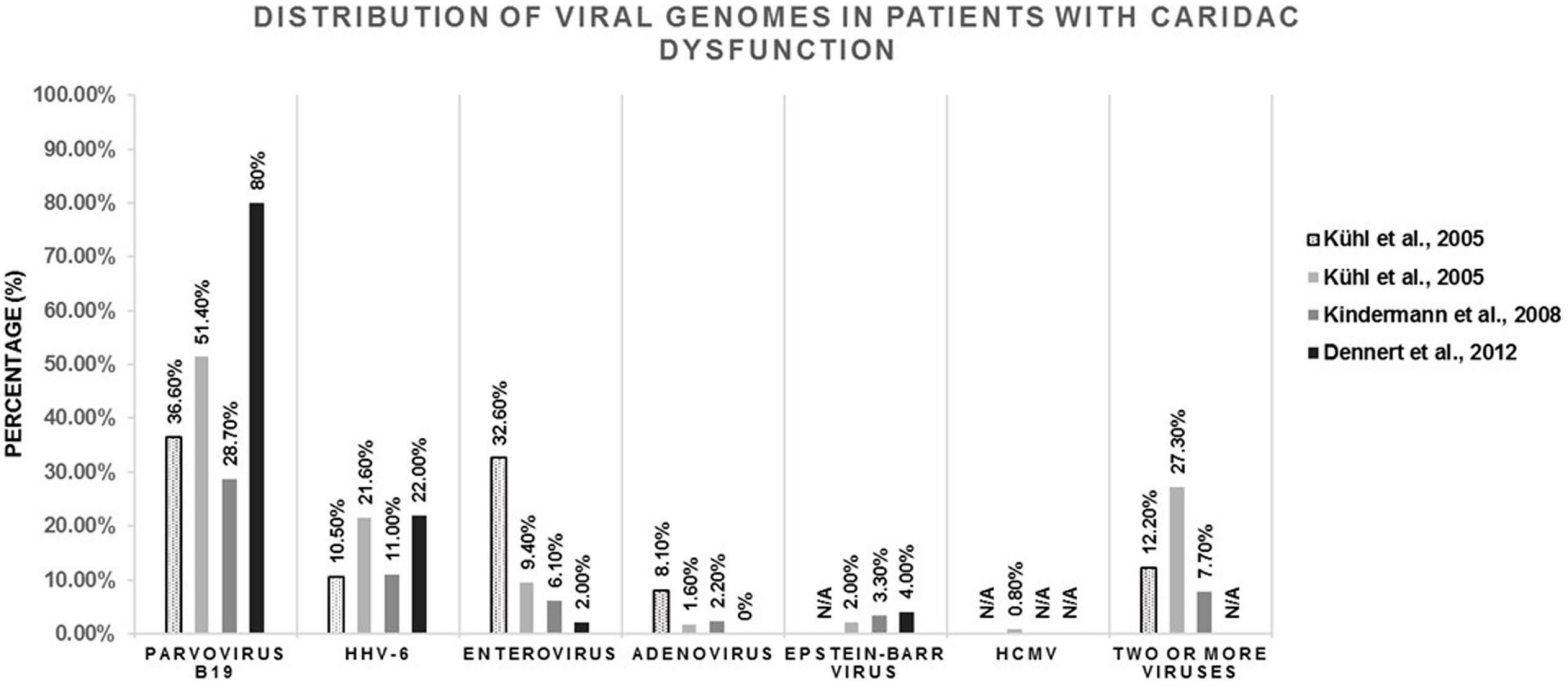
Analysis of viral genome presence in human endomyocardial samples obtained from patients with cardiomyopathy (dilated cardiomyopathy or myocarditis), as described by Kühl et al. (2005a,b), Kindermann et al. (2008), and Dennert et al. (2012). The amount of parvovirus B19, HHV-6, Enterovirus, Adenovirus, Epstein-Barr virus, HCMV or multi-viral presence (two of more viruses present in one endomyocardial sample) found in each individual study is shown as a percentage (%) in the respective population.

Viral persistence in the human heart tissue is a result of an infection that was not fully cleared by the host’s immune system. Depending on their outcome, persistent infections are divided into latent and chronic subcategories, where the latent infection lasts for a life time of a host and it is characterized by periods of quiescence and reactivation of viral replication, whereas the chronic infection, described by continuous viral presence, may be eventually eradicated from the host’s cells. Studies focusing on patients presenting cardiomyopathic symptoms showed that parvovirus B19, adenovirus, CVB3, Epstein–Barr virus, human cytomegalovirus and human herpesvirus 6 are primarily detected in heart samples exhibiting signs of infection (Figure 2). In a study described by Bowles et al, samples obtained from 624 myocarditis patients in the United States were analyzed for the presence of adenovirus, enterovirus, parvovirus B19, influenza A, herpes simplex virus, cytomegalovirus and respiratory syncytial virus (Bowles et al., 2003). Viral genome was detected in 38% of patients, and 93% of these patients had a prior history of viral infection, whereas only 3 out of 215 control samples derived from patients with no recent history of viral infection or myocarditis were positive for viral genome (Bowles et al., 2003). It was also reported that viral RNA could be detected in 46.4% of clinically diagnosed group of 69 heart failure patients in Canada (Hanson et al., 2022). On the other hand, 20% of patients suffering from DCM were positive for viral genomes in their heart samples, and 7% of these patients had a confirmed diagnosis of prior viral infection (Bowles et al., 2003), suggesting that not all viral infections are successfully eliminated, which may lead to low grade levels of inflammation, engagement of immune system, or, in some cases, disease. Aside from active viral infection that causes acute cardiac inflammation, viruses have also been identified in heart tissue autopsy samples derived from patients with no prior history of heart complications. Therefore, it remains to be determined if these viral genomes are bystanders that neither initiate nor contribute to already existing heart pathology, or if they are involved in cardiac disease process.

## Parvovirus B19

Parvovirus B19 (B19V), a small single-stranded DNA virus that belongs to the *Parvoviridae* family, is frequently found in patients’ heart tissue samples, regardless of disease status. The virus causes a common childhood infection, known as erythema infectiosum, or fifth disease, which presents as a rash. In the majority of cases, the disease is mild, although complications, such as anemia, may develop in individuals with a compromised immune system (Serjeant et al., 2001; Rajput et al., 2012). It is estimated that by the age of 50, over 50% of individuals have antibodies specific to B19V, confirming its prevalence in a population. Many studies identified B19V as a causative agent of acute myocarditis (Schowengerdt et al., 1997; Jain et al., 2013; Ackermann et al., 2020). Nonetheless, B19V does not infect myocytes, but instead, it targets the endothelial cells of myocardial vessels (Kandolf, 2004; Mahrholdt et al., 2006). The subsequent recruitment of inflammatory cells causes myocyte damage (Kandolf, 2004), which negatively impacts the function of the cardiac muscle. Indeed, studies reported inflammatory infiltrates and an altered architecture of the heart muscle fibers in a patient suffering from fulminant myocarditis (Ackermann et al., 2020), and lymphocyte infiltration accompanied by myocyte vacuolization and myocardial fibrosis in a case of B19V-induced acute heart failure (Alberti et al., 2012). In addition to acute cases of myocarditis, the virus has also been found in patients suffering from cardiomyopathies and heart failure, and also in EMB samples derived from patients with no prior heart disease. Whether B19V is a harmless bystander, or a causative agent that may induce heart damage, still remains to be answered.

B19V persistence in the cardiac muscle and its potential impact on heart disease has been a subject of many studies in the past. When assessing the prevalence of B19V in patients exhibiting chronic heart failure (CHF), it was revealed that 31 out of 37 (84%) individuals with CHF accompanied by diastolic dysfunction had B19V genome in their EMB samples, compared to 7 out of 33 (21%) patients with no diastolic dysfunction (Tschöpe et al., 2005), suggesting that the endothelial impairment caused by the virus may contribute to the development of the disease. B19V persistence after the initial infection has been also linked to atypical angina pectoris (Yilmaz et al., 2008). During the course of infection, B19V targets cardiac epithelial cells, which leads to their apoptosis. Endothelial microparticles, which are released during this process, are known to negatively affect vascular homeostasis (Yilmaz et al., 2008; Bachelier et al., 2017). Patients suffering from myocarditis and positive for B19V virus in their heart tissues had elevated levels of circulating endothelial microparticles compared to individuals with myocarditis, but no detectable B19V genomes (Bachelier et al., 2017). Similar results were observed in a mouse model of B19V myocarditis, which is consistent with endothelial damage induced by the virus.

Even though B19V genomes are commonly seen in endomyocardial tissues associated with heart disease, B19V persistence has been also observed in healthy individuals. In a comparative study conducted in the Netherlands, Dennert et al reported the presence of the viral genome in 100 out of 125 (80%) patients diagnosed with DCM and in 15 out of 20 (75%) control individuals with no heart disease (Dennert et al., 2012). Similarly, in a study performed by Bültmann et al, 4 out of 26 (15%) of women with peripartum cardiomyopathy and 5 out of 33 (15%) of control individuals had B19V genome detected in their EMB samples (Bültmann et al., 2005). When examining B19V viral loads, no significant differences were reported between patients with heart disease and controls (Schenk et al., 2009), suggesting that the amount of virus in the heart tissue cannot be used to predict heart disease. Recently, Bironaitė et al reported that chronic persistence of B19V in the myocardium was not correlated with lymphocyte infiltration; however, the heart tissue samples positive for the viral genome exhibited mitochondrial impairment (Bironaitė et al., 2022), suggesting that another mechanism may be behind B19V-induced heart injury. The inflammatory processes that occur in the aftermath of viral infection can also induce tissue damage. The NS1 protein of B19V is known to cause apoptosis of endothelial cells, and it also stimulates the expression of pro-inflammatory cytokines (Duechting et al., 2008). Expression of hPVB19 NS1 protein in HEK-293 T cells triggers an upregulation of some pro-inflammatory cytokines, such as IL-6 and TNF-ɑ (Jalali et al., 2022). Therefore, the potential presence of viral proteins in the heart tissue, which could be a result of delayed clearance, may contribute to chronic inflammation, which in turn could negatively impact the heart function. Furthermore, the assessment of viral activity could greatly impact the clinical interpretation of potential B19V genome finding in a patient’s heart tissue. A study conducted in Germany by Pietsch et al reported that 403 out of 576 (70%) of patients diagnosed with unexplained heart failure had B19V genome in their heart tissues, yet viral mRNA was detected in 26.9% of the analyzed samples (Pietsch et al., 2020). It will be beneficial to examine the degree of cardiac damage and the level of inflammation in individuals with viral activity to uncover the link between viral replication and cardiac disease. In the future, it will also be critical to assess if B19V may play a role in myocardium remodeling through potential impairment of gene expression, and to assess the role of viral proteins in the cardiac muscle damage.

## Coxsackieviruses

Among enteroviruses, Coxsackie B viruses (CBVs) are one of the most common causes of cardiac inflammation (Gaaloul et al., 2014). This common infection usually causes flu-like symptoms that include fever, rash, joint pain, and sometimes gastrointestinal distress; yet, unresolved CBV infection can negatively impact the heart muscle. In fact, between 25 and 40% of myocarditis or cardiomyopathy cases are attributed to CBVs in children and young adults (Leonard, 2004). Like most cardiotropic viruses, CBVs can cause inflammation of the heart in two ways: direct cardiac injury or indirect immune mediated damage. CBVs utilize a trans-membrane protein, called coxsackievirus and adenovirus receptor (CAR), and a co-receptor called decay accelerating factor (DAF) for entry. A mouse model system for CVB3 induced myocarditis has been utilized for over 60 years (Grodums and Dempster, 1959), and it was shown that CAR deficiency prevents CVB3 infection and the subsequent development of myocarditis (Shi et al., 2009). Once internalized, the positive sense single-stranded RNA genome is translated into a single polyprotein, eventually cleaved by viral proteases to produce proteins necessary to replicate, package viral genome, and then exit cells *via* lysis. These proteases also cleave host factors to facilitate viral replication and dampen the immune response (Mukherjee et al., 2011). In an attempt to inactivate the immune system and enhance virion release, CBVs cleave host translation initiation factors, cease interferon production, disrupt calcium signaling, endoplasmic reticulum function and other host cell machineries; all contributing to a direct impact on normal cardiac function and physiology (Massilamany et al., 2014). On the other hand, the immune mediated damage is initiated by recognition of viral RNA by the host pattern recognition receptors (PPRs), which promotes transcription of pro-inflammatory cytokines, including interleukin 1α (IL-1α), IL-1β, IL-6, tumor necrosis factor-α (TNF-α) and IFNɣ (Henke et al., 1950). The immune mediated damage is a result of a cytokine storm, which can alter both infected and healthy cardiac cells, as well as the production of cardiac autoantibodies (Henke et al., 1950, 1991). From there, the infection can either recede/resolve, or develop into a persistent cardiac inflammation simultaneously mediated by cytokine response and autoimmune response against cardiac protein (Henke et al., 1991; Massilamany et al., 2014).

Acute myocarditis cases associated with enterovirus infection have been described in the past (Chapman et al., 2008; Wu et al., 2013). Mehta et al reported an acute case of CVB3 heart infection with left ventricular failure accompanied by secondary pulmonary arterial hypertension (Mehta et al., 2018). The pathology of the heart tissue, confirmed by EMB, revealed the presence of lymphocytic infiltrates with myocyte necrosis, along with the presence of T cells (Mehta et al., 2018). Interestingly, previous infections can also play a role in the development of cardiac inflammation. A patient with a biopsy-proven myocarditis induced by CVB3 was reported to develop acute pericarditis months after the initial disease onset, suggesting that chronic or latent CVB3 infections may pose a long-term threat to cardiac health (Wessely et al., 2004).

The persistence of enterovirus in the heart tissue and its potential role in cardiomyopathy has been an active area of investigation for decades. The analysis of human EMB samples showed that 25 out of 70 (37.5%) of patients with idiopathic DCM and 21 out of 70 (32.8%) of patients with chronic coronary disease had detectable levels of enterovirus, whereas no EV genome was picked up in the hearts of 45 healthy donors (Andréoletti et al., 2000). Only 2 out of 70 individuals had an RNA replication intermediate, known as antigenomic RNA, present in their samples along with VP1 antigen expression, consistent with active viral replication. The majority of patients (97%) had a latent EV infection, demonstrated by the absence of antigenomic RNA at the time of heart tissue collection (Andréoletti et al., 2000). Evaluation of 172 patients with biopsy-proven viral infection in Germany also reported the presence of EV genome in 56 out of 172 (32.6%) of these cases (Kühl et al., 2005a). In addition, 23 out of 245 (9.4%) patients with idiopathic left ventricular dysfunction were positive for EV genome (Kühl et al., 2005b). On the other hand, only 2% of patients (*n* = 125) with idiopathic DCM were positive for the EV genome in a study by Dennert et al in the Netherlands (Dennert et al., 2012).

A piece of evidence that may link CVB persistence to cardiac inflammation comes from a fatal case of myocarditis patient. The CVB2 genome, identified as a causative agent of myocarditis, had naturally occurring deletions within the cloverleaf motif (1–22 and 1–25 nucleotide deletions; Oka et al., 2005). These specific mutant viruses did not induce cytopathic effect in a mouse and human cardiac tissue, which is consistent with CVB2 persistence (Kim et al., 2005; Chapman et al., 2008; Smithee et al., 2015). Furthermore, Bouin et al reported persistent populations of CVB3 containing 15–48 deletions in the 5’UTR in human endomyocardial tissues (Bouin et al., 2016). It has been postulated that the lower replicative capacity of the virus harboring these deletions may enable its persistence in the cardiac muscle, and/or contribute to wild type CVB3 genomic recombination (Holmblat et al., 2014; Bouin et al., 2016). In addition, an analysis of heart tissue samples obtained from patients suffering from DCM revealed the presence of persistent Enterovirus B viral infection, and the identified viral species contained genomic deletions in the 5’ UTR region (Bouin et al., 2019). Transfecting primary human cardiomyocytes with viral mRNAs containing these deletions resulted in viral replication, as demonstrated by the analysis of total viral RNA levels until 48 h post transfection, but no formation of viral plaques (Bouin et al., 2019). Interestingly, when combined with full-length viral mRNAs, these 5’ UTR deleted variants could impair cardiomyocytes with the help of viral proteinase 2A proteolytic activity (Bouin et al., 2019). Although this remains to be experimentally determined, these findings may suggest that viral deletions or mutations could contribute to, or induce, viral persistence. For instance, the naturally occurring genetic alterations could help a virus escape the detection of the host immune system, or aid in the replication of wild type viral species. These mutations could also impact the viral/host protein binding. At the same time, the response of the immune system to CVB infection can also damage the heart. The release of Natural Killer (NK) cells has been shown to disrupt the myocardial cytoskeleton (Young, 1990), and the subsequent recruitment of T lymphocytes further propagates myocardial damage (Pollack et al., 2015). Together, these factors could help a virus persist in the cardiac tissue and damage the cardiac muscle. In the absence of viral clearance, DCM may ensue. In humans, assessing the mechanistic details involved in virus-induced disease pathology has been difficult due to limited sample availability. However, animal studies support the involvement of viruses in the disease process. Specifically, a mouse model system of CVB3- induced heart inflammation has provided evidence for viral involvement in the disease process. For example, the presence of replication-restricted CVB3 genome in the myocytes leads to the development of DCM in mice, and the overall disease process is similar to that described in humans, including myocytes damage, impairment of systolic function and cardiac muscle remodeling (Wessely et al., 2004).

## Herpesviruses

Epstein–Barr virus (EBV), human herpesvirus 6 (HHV-6) and human cytomegalovirus (HCMV), which are members of the *Herpesviridae* family, have been previously linked to myocarditis (Ishikawa et al., 2005; Leveque et al., 2011; Goodrum et al., 2012; Saraca et al., 2018). These double stranded DNA viruses are prevalent among the population, and it is estimated that over 90% of people worldwide have had an EBV infection, whereas more than 50% of the population have been infected with HHV-6 and HCMV (Emery and Clark, 2007; Dunmire et al., 2018). After the initial infection these viruses persist in the human body, yet there is no virus production because of limited viral gene expression (Goodrum et al., 2012; Kerr, 2019). Circumstances that favor viral reactivation, such as lower immune system function, or disease, may reverse the latent stage, which leads to the production of viral progeny. Although uncommon, EBV has been shown to cause myocarditis in patients (Ishikawa et al., 2005; Mutlu et al., 2011). An acute case of EBV-induced heart inflammation has been previously associated with the presence of lymphocytic infiltrates and a T cell marker CD_45_R0 (Ishikawa et al., 2005). The disease severity progression and the outcome vary among patients, and in some cases symptoms can resolve on their own, whereas in others the heart becomes irreversibly damaged, resulting in a patient’s death (Ishikawa et al., 2005; Antonakaki et al., 2016). Reactivation of the virus after the initial infection has also been previously reported, as shown by the presence of EBV-encoded RNA and mononuclear infiltrates in the pericardium (Richter et al., 2013). Similarly, cases of HCMV-induced heart inflammation have been previously described (Vanstechelman and Vandekerckhove, 2012; Saraca et al., 2018). Interestingly, neither EBV nor HCMV can infect cardiomyocytes due to the lack of specific receptor binding. Instead, these two viruses were previously detected in cardiac inflammatory cells (T-cells, B-cells and macrophages; Pankuweit and Klingel, 2013), suggesting that the cardiac damage that occurs during the course of viral infection may be indirectly triggered by the immune system activation.

The prevalence of HHV-6 genome in human heart samples is the highest within the *Herpesviridae* family. Many cases of HHV-6 induced myocarditis have been described in the context of a host’s immunocompetent state (Fukae et al., 2000; Leveque et al., 2011), and also as a co-infection agent (Fukae et al., 2000). HHV-6 is also an important agent thought to be involved in pediatric myocarditis, with possible role in DCM (Reddy et al., 2017). This is not surprising, as an HHV-6 infection, also known as roseola, is prevalent in young children. An acute case of pediatric myocarditis that occurred after an HHV-6 infection revealed mononuclear cells and neutrophils infiltrates in the heart, along with myocardial cell degeneration (Yoshikawa et al., 2001). Another study described elevated levels of cardiac damage markers, such as troponin, and increased levels of inflammatory markers, in the aftermath of HHV-6 infection of the heart (Reddy et al., 2017). It has been suggested that HHV-6 may cause endothelial and diastolic dysfunction through direct infection of vascular endothelial cells (Kühl et al., 2005b; Reddy et al., 2017).

Although the exact mechanism behind the long-term exposure of the cardiac muscle to the abovementioned herpesviruses needs to be elucidated, current evidence suggests that such association has negative consequences for the function of the heart. In particular, EBV and HHV-6 have been found in EMB samples derived from individuals suffering from heart disease, or obtained during heart transplant. A study conducted in Germany by Kindermann et al showed the presence of EBV in 6 out of 181 (3.3%) EMB samples obtained from individuals with clinically suspected myocarditis (Kindermann et al., 2008). On the other hand, 20 out of 181 (11%) of patients were positive for HHV-6 genome. An analysis of a group of patients diagnosed with idiopathic DCM by Dennert et al in the Netherlands showed the presence of EBV and HHV-6 genomes in 4 and 22% of patients (n = 125), respectively (Dennert et al., 2012). On the contrary, heart tissue samples derived from control population did not have any detectable levels of these genomes. A higher percentage of heart tissue samples positive for HHV-6 was also detected by Kühl et al, where 23 out of 245 (9.4%) patients with presumptive DCM had HHV-6 genome in their hearts, but only 5 out of 245 (2%) were positive for EBV, and 2 out of 245 (0.8%) had HCMV genome (Kühl et al., 2005b). Low levels of HHV-6 DNA were detected in 79 out of 756 (10.4%) patients with unexplained heart failure (HF) and in 29 out of 354 (8%) patients without the disease in a study performed in Germany by Elsanhoury et al (Elsanhoury et al., 2022). About 62% of patients with unexplained HF had myocardial inflammation, suggesting that the presence of HHV-6 is not always associated with an inflammatory state (Elsanhoury et al., 2022). A study by Mahrholdt et al conducted in Germany evaluated the potential correlation between myocardial damage and the presence of HHV-6 (Mahrholdt et al., 2006). The virus was found in 16 out of 87 (18.3%) patients suffering from clinically proven myocarditis, and half of these patients also exhibited symptoms of heart failure. Interestingly, if a patient was positive for B19V and HHV-6 viral genomes, they had more severe heart failure symptoms at the time of diagnosis and during a follow-up, suggesting that the presence of multiple viruses may further impact the function of the heart. The negative consequences associated with the persistence of HHV-6 in the human heart tissue were further confirmed by Escher et al, who analyzed left ventricular ejection fraction (LV-EF) in patients positive for HHV-6 A and B infections over a course of time. It was revealed that persistence of HHV-6 B in the heart tissue at follow up was linked to cardiac dysfunction, whereas a resolved infection improved the function of the heart (Escher et al., 2015).

Furthermore, the persistence of herpesviruses was previously associated with viral protein presence. Laser capture microdissection combined with confocal microscopy was used to determine the localization of EBV in the hearts of patients with inflammatory cardiomyopathy. EBNA-1 protein, which is important for EBV persistence, was shown to localize to nuclei of a few myocytes derived from patients with cardiomyopathy (Chimenti et al., 2004). In such case, one may speculate that the presence of EBV protein in the heart tissue could cause myocyte damage, which could in turn impact the cardiac muscle architecture. In the future, it will be important to determine if other EBV proteins could localize to myocytes. Furthermore, given that the majority of the world’s population is seropositive for EBV, it will be beneficial to determine if the latent virus, which resides in B lymphocytes, could have any impact on heart disease upon reactivation.

## Adenovirus

Adenovirus is primarily responsible for respiratory track symptoms associated with common cold, but on rare occasions, the virus can cause inflammatory cardiomyopathy and/or myocarditis in adults and children (Martin et al., 1994; Kühl et al., 2005a; Treacy et al., 2010). Similar to CVB3, adenovirus can directly infect cardiomyocytes by attaching to CAR (coxsackievirus and adenovirus receptor), and the resulting infection causes cardiomyocyte damage and cytoskeletal disruption (Tschöpe et al., 2021). Adenovirus type 8 was detected in 17.4% of patients (*n* = 23) suffering from acute idiopathic DCM, which was severe enough to require a heart transplant (Hosseini et al., 2018). A recent study by Hanson et al in Canada revealed that adenovirus was detected in 19 out of 69 (27.5%) patients suffering from heart failure (Hanson et al., 2022). A study by Bowles et al conducted in the United States showed that 142 out of 624 (23%) patients with myocarditis, and 18 out of 149 (12%) patients with DCM had adenovirus in their heart tissues (Bowles et al., 2003). Similarly, 12 out of 94 (12.7%) patients with LV dysfunction were positive for adenovirus genome in their heart samples, whereas no viral genome was detected in the control population (Pauschinger et al., 1999). Interestingly, the patients enrolled in the study were not diagnosed with active myocarditis, which could suggest that the virus may have persisted in the cardiac tissue after the initial infection. Earlier studies showed apoptosis in cardiomyocytes chronically infected with adenovirus (Bowles et al., 1997). It is known that two adenovirus proteins: E1A and E1 play a role in inducing apoptosis; therefore, future studies may uncover the mechanistic aspects of cardiomyocyte damage during adenovirus infection.

## SARS-CoV-2

SARS-CoV-2, the virus responsible for the coronavirus disease 2019 (COVID-19), is known to target lungs and initiate a wide range of respiratory symptoms (Yi et al., 2020). Nonetheless, during the course of the global COVID-19 pandemic, it quickly became apparent that other systems, including the cardiovascular system, are also prone to damage. In fact, some patients suffering from COVID-19 infection experience irregular heartbeat, cellular injury, myocardial interstitial fibrosis, or myocarditis (Egas et al., 2021; Farshidfar et al., 2021). Up to 30% of hospitalized patients are at risk of developing myocarditis, and their long-term outcomes are worse compared to patients without cardiac involvement (Shchendrygina et al., 2021; Jumah et al., 2022). It is thought that SARS-CoV-2 can directly infect the heart (Bose and McCarthy, 2020), with the Ace2 receptor serving as an entry point to cardiomyocytes. Interestingly, SARS-CoV-2 infection causes the internalization of the Ace2 receptor, which diminishes its expression in the cells of the heart (Li et al., 2020). This has been proposed to further enhance the risk of cardiovascular disease because the loss of Ace2 is associated with heart failure (Patel et al., 2016). It has been also suggested that the virus can infect the vascular endothelial cells, resulting in cardiovascular damage (Liu et al., 2021).

The analysis of post-mortem human LV myocardium derived from patients clinically diagnosed with COVID-19 infection and myocarditis showed cardiomyocyte necrosis (Bailey et al., 2021). SARS-CoV-2 nucleocapsid proteins have also been shown to localize to cardiomyocytes of infected patients (Bailey et al., 2021). Furthermore, assessment of cardiac damage associated with the COVID-19 infection revealed unique cardiac features, such as non-myocarditic inflammation, single myocyte necrosis, or fibrosis (Fox et al., 2020; Halushka and Vander Heide, 2021; Jumah et al., 2022). However, other studies demonstrated that COVID-19 induced myocardial injury may occur in response to cytokine release storm (CRS), also known as cytokine storm (Lawal et al., 2022). This hyper-inflammatory state initiated by the host’s immune response system can directly trigger the release of cytokines, and COVID-19 patients exhibited increased levels of troponin, myoglobin, C-reactive protein and interleukin-6 (IL-6; Ruan et al., 2020). Another study suggested that elevated circulating levels of IL-6 and TNF-ɑ during COVID-19 infection are correlated with increased disease severity (Del Valle et al., 2020).

The persistence of COVID-19 disease symptoms in some patients is now well documented, which is a phenomenon referred to as Long COVID-19, or Post-acute sequelae of COVID-19 (PASC; Proal and VanElzakker, 2021). A study performed in Denmark by Vibholm et al reported that 12% of individuals were positive for SARS-CoV-2 genome 23 days after recovery (*n* = 203), whereas over 5% of patients still had detectable viral genome 90 days post recovery (Vibholm et al., 2021). Unsurprisingly, the risk of cardiovascular events is not only associated with an ongoing infection, but it persists over time. Xie et al reported increased rates of myocarditis and pericarditis, as well as ischemic cardiomyopathy 12 months post-acute COVID-19 incident (Xie et al., 2022). Although the molecular mechanisms that dictate the potential development of cardiac disease in the aftermath of COVID-19 infection are yet to be elucidated, it has been suggested that the response of the host’s immune system may be involved. For example, patients with an acute COVID-19 infection present diminished levels of dendritic cells, and this trend may persist in some individuals for up to 7 months (Pérez-Gómez et al., 2021). Because dendritic cells are crucial for the proper functioning of the innate immune system, their altered levels may negatively impact the host response to viral infection. Future studies will be important to aid in our understanding of long-term cardiovascular consequences associated with SARS-CoV-2, and that knowledge will impact patients’ outcomes.

## Discussion

Heart disease is one of the most common causes of death worldwide. Genetic, environmental or pathogenic factors may contribute to the development and/or progression of cardiac disease, and viral infection, followed by the inflammation of the heart muscle, is a well-recognized phenomenon that can impair the heart. The acute form of the disease can be directly linked to viral infection through confirmation of viral genome presence, histological examination of the heart tissue, and clinical symptoms. However, it is not clear if the long-term presence of viral genomes in the human heart tissue may trigger a similar effect over time. This question is particularly important considering that viral genomes are routinely discovered in heart samples of healthy individuals in the absence of clinical symptoms.

Accumulating evidence suggests that under certain circumstances, viral genomes or viral proteins, may promote an inflammatory state that could disrupt the function of the cardiac cells. A time-course analysis of long-term consequences associated with biopsy-proven myocarditis and idiopathic DCM suggest that virus clearance correlates with left ventricular ejection fraction (LVEF) improvement, and that phenomenon is true for any type of a virus (Kühl et al., 2005a). On the other hand, viral persistence in the heart tissue is associated with a worse LV prognosis. In the future, it will be important to elucidate the common themes associated with any virus species persistence in the heart tissue, as it may help with the development of broad-range therapies for people suffering from virus-induced inflammation.

Viral proteins should also be examined for their potential role in heart disease. Transgenic mice that express EBNA-LP, a viral gene thought to play a role in B-cell transformation associated with EBV infection, displayed dilated ventricles and enlargement of the left atrium, which ultimately caused death of the animals (Huen et al., 1993). The EBNA-LP gene expression was also detected in the heart of the animals. Interestingly, it took several months for the transgenic animals to develop heart failure, and no symptoms presented before the disease onset. This result implies that active viral replication may not be needed to induce potential heart damage. Studies performed by Binkley et al showed that an enzyme encoded by EBV, deoxyuridine triphosphate nucleotidohydrolase (dUTPase), triggers the production of pro-inflammatory cytokines and the expression of intracellular adhesion molecule-1 (ICAM-1) in peripheral blood monocytes (Binkley et al., 2013). dUTPase, an early EBV protein released during viral reactivation, may therefore be a contributing factor in endothelial damage and progression of coronary artery disease.

Finally, viral persistence is strongly dependent on the function of the host’s immune system. Even if the virus is not eliminated from the host in a timely manner, the properly functioning immune system prevents or limits subsequent disease outbreaks; yet that changes if the immune system is weakened or out of balance. Furthermore, a viral infection could aid in reactivation of other viral species already residing in the heart through immune system impairment. A recent report described that SARS-CoV-2-induced infection may lead to reactivation of EBV (García-Martínez et al., 2020; Chen et al., 2021). Other viruses that belong to the Herpesvirus family: HHV-6 and HHV-7 were also shown to be reactivated in patients infected with SARS-CoV-2 (Drago et al., 2021). It was speculated that these events may have been initiated by SARS-CoV-2 misregulation of the host interferon response, which is crucial in the fight against pathogens (Acharya et al., 2020). Whether a similar molecular mechanism may be responsible for reactivation of persistent viruses in cardiac tissue remains to be elucidated. Nonetheless, when assessing cardiac inflammation, it is important to consider that many viral species, both from past and on-going infection events, could be the driving force behind cardiac damage.

## Author contributions

All authors have made a substantial intellectual contribution to the work. All authors contributed to the article and approved the submitted version.

## Funding

This research is supported by the Faculty Research Fund from the University of New Haven (AK).

## Conflict of interest

The authors declare that the research was conducted in the absence of any commercial or financial relationships that could be construed as a potential conflict of interest.

## Publisher’s note

All claims expressed in this article are solely those of the authors and do not necessarily represent those of their affiliated organizations, or those of the publisher, the editors and the reviewers. Any product that may be evaluated in this article, or claim that may be made by its manufacturer, is not guaranteed or endorsed by the publisher.
